# Serological assays to measure dimeric IgA antibodies in SARS‐CoV‐2 infections

**DOI:** 10.1111/imcb.12682

**Published:** 2023-08-18

**Authors:** Zihui Wei, Fiona Angrisano, Emily M Eriksson, Ramin Mazhari, Huy Van, Shuning Zheng, Rob J Center, Irene Boo, James McMahon, Jillian Lau, Nicholas Kiernan‐Walker, Shazia Ruybal‐Pesántez, Ivo Mueller, Leanne J Robinson, David A Anderson, Heidi E Drummer

**Affiliations:** ^1^ Burnet Institute, 85 Commercial Road Department of Life Sciences Melbourne VIC 3004 Australia; ^2^ Walter and Eliza Hall Institute of Medical Research Department of Population Health and Immunity Parkville VIC 3052 Australia; ^3^ The University of Melbourne Department of Medical Biology Parkville VIC 3052 Australia; ^4^ Peter Doherty Institute for Infection and Immunity at The University of Melbourne Parkville VIC 3052 Australia; ^5^ Monash University Department of Infectious Diseases Alfred Health Melbourne VIC 3004 Australia; ^6^ Monash University Department of Microbiology Clayton VIC 3168 Australia

**Keywords:** COVID‐19, dIgA, mucosal immunity, serological assay

## Abstract

Current serological tests cannot differentiate between total immunoglobulin A (IgA) and dimeric IgA (dIgA) associated with mucosal immunity. Here, we describe two new assays, dIgA‐ELISA and dIgA‐multiplex bead assay (MBA), that utilize the preferential binding of dIgA to a chimeric form of secretory component, allowing the differentiation between dIgA and monomeric IgA. dIgA responses elicited through severe acute respiratory syndrome coronavirus 2 (SARS‐CoV‐2) infection were measured in (i) a longitudinal panel, consisting of 74 samples (*n* = 20 individuals) from hospitalized cases of coronavirus disease 2019 (COVID‐19); (ii) a longitudinal panel, consisting of 96 samples (*n* = 10 individuals) from individuals with mild COVID‐19; (iii) a cross‐sectional panel with PCR‐confirmed SARS‐CoV‐2 infection with mild COVID‐19 (*n* = 199) and (iv) pre–COVID‐19 samples (*n* = 200). The dIgA‐ELISA and dIgA‐MBA demonstrated a specificity for dIgA of 99% and 98.5%, respectively. Analysis of dIgA responses in the longitudinal panels revealed that 70% (ELISA) and 50% (MBA) of patients elicited a dIgA response by day 20 after PCR diagnosis with a SARS‐CoV‐2 infection. Individuals with mild COVID‐19 displayed increased levels of dIgA within the first 3 weeks after diagnosis but responses appeared to be short lived, compared with sustained IgA levels. However, in samples from hospitalized patients with COVID‐19 we observed high and sustained levels of dIgA, up to 245 days after PCR diagnosis. Our results suggest that severe COVID‐19 infections are associated with sustained levels of plasma dIgA compared with mild cases.

## Graphical abstract

Current serological tests cannot differentiate between total immunoglobulin A and dimeric immunoglobulin A (dIgA) associated with mucosal immunity. We have developed two new assays based on the preferential binding of dIgA to a chimeric form of secretory component that differentiates between dIgA and monomeric IgA. The use of a chimeric form of secretory component allows the specific measurement of dIgA in the immune system elicited through severe acute respiratory syndrome coronavirus 2 (SARS‐CoV‐2) infection and our results suggest that severe coronavirus disease 2019 (COVID‐19) is associated with more sustained levels of dIgA in plasma compared with mild cases.

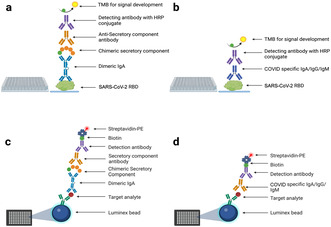

## INTRODUCTION

Severe acute respiratory syndrome coronavirus 2 (SARS‐CoV‐2) is a virus that causes a multisystem infection known as coronavirus disease 2019 (COVID‐19).^1^ Epithelial cells located in the nasopharynx are infected when the virus receptor‐binding domain (RBD) interacts with the angiotensin‐converting enzyme‐2 receptor.^2^ Respiratory infections, including SARS coronaviruses, trigger the production of immunoglobulin (Ig)M, IgG and IgA, which can all be measured in serological assays. SARS‐CoV‐2 infection induces a mucosal response that involves the secretion of IgA. Dimeric IgA (dIgA) produced locally at mucosal‐associated lymphoid tissue is transported across mucosal surfaces *via* the interaction with the basolaterally located polymeric Ig receptor. After binding the polymeric Ig receptor, dIgA is transcytosed across the mucosal surface to the apical plasma membrane, with concomitant formation of a disulfide bond between dIgA and polymeric Ig receptor. Polymeric Ig receptor is then cleaved to form the secretory component (SC), and secretory IgA (SIgA) is subsequently released into secretions such as saliva, tears and mucous.^3^

SIgA and dIgA play numerous roles in protecting hosts, including neutralizing toxins and pathogens, preventing adhesion of pathogens and facilitating clearance of foreign antigens.^4^, ^5^ It has been previously demonstrated that IgA is more effective than IgG in preventing influenza infections in both murine and human models.^6^, ^7^ Despite the importance of dIgA and SIgA in host defense, their roles have not been well studied. Moreover, conventional IgA serology currently does not discriminate monomeric IgA from dIgA. Existing serological tests are limited to measuring total IgA and in the absence of evidence to the contrary, it is commonly assumed that plasma IgA and dIgA originate from the same B cells and not distinct populations.

Work by our team indicates that dIgA can be detected by a recombinant chimeric human SC.^8^, ^9^ The human SC contains five subdomains, D1 to D5, and binds to both IgM and dIgA subclasses. We previously developed and characterized a chimera of the human SC, where D1 was replaced with the equivalent domain of rabbit SC and D2–D5 are from human SC. The resulting chimeric form of SC (CSC) reagent preferentially binds dIgA and has greatly reduced capacity to bind IgM.^8^ The preferential binding of CSC to dIgA enables the distinction between monomeric IgA and dIgA in plasma and the detection of the role of dIgA responses in mucosal immunity.^8^

Here, we describe two novel serological assays developed to examine the temporal production of dIgA and the development of mucosal immunity in SARS‐CoV‐2 infection. Using modified ELISA and multiplex bead assay (MBA), we tracked IgG, IgA and dIgA responses in SARS‐CoV‐2–infected patients with differing degrees of COVID‐19 severity. We measured specific antibody levels longitudinally in plasma and explored the use of dIgA as a biomarker of recent infection and the development of mucosal immunity. Our results demonstrate that dIgA antibodies are detectable in humans infected with COVID‐19 before SARS‐CoV‐2–specific IgA, suggesting dIgA could play a role in the early stages of the immune response to SARS‐CoV‐2 infection.

## RESULTS

### Development of ELISA and MBA to measure dIgA SARS‐CoV‐2

An immobilized recombinant SARS‐CoV‐2 RBD antigen (either bound to ELISA plates or coupled to carboxylated magnetic beads) was used to bind immunoglobulins preincubated with CSC reagent. Antigen‐specific dIgA was then detected by an anti‐SC antibody followed by a secondary antibody for quantitation (Supplementary figure 1a and c). To validate these assays, we utilized monoclonal IgG, IgA, IgM and dIgA antibodies specific to the SARS‐CoV‐2 RBD protein (BetaCoV/Australia/VIC/01/2020). We engineered recombinant monoclonal antibodies by chimerizing the heavy‐chain antigen‐binding fragment of monoclonal antibody CB6 with the Fc portion of IgA1, IgA2, IgG or IgM (Supplementary figure 2a).^8^ To produce dIgA1 and dIgA2 or IgM proteins, cells were transfected with vectors encoding the CB6 kappa light chain and the CB6 IgA1, IgA2 or IgM chimeric heavy chain plus the J chain. Alternatively, monomeric forms of chimeric monomeric CB6 IgA1 (mIgA1) and IgA2 (mIgA2) were produced in the same way in the absence of the J chain. CB6 IgG was produced by transfection of cells with the IgG heavy chain and the kappa light chain (Supplementary figure 2b and c). dIgA1 and dIgA2, mIgA1 and mIgA2 and IgM were purified using protein L agarose beads. IgG was purified using protein G agarose beads. Reducing sodium dodecyl sulfate–polyacrylamide gel electrophoresis confirms the presence of heavy and light chains for each antibody subclass, and nonreducing sodium dodecyl sulfate–polyacrylamide gel electrophoresis confirms the formation of dIgA1 and dIgA2 species migrating at > 250 kDa (Supplementary figure 2d). In the case of IgM, only a small amount of IgM failed to migrate into the gel likely to be higher‐order forms of IgM. Analytical size‐exclusion chromatography was used to verify that the IgA1 and IgA2 had dimerized when coexpressed with the J chain and a small amount of higher‐order IgM, possibly pentameric, was present in protein L–purified proteins. The presence of heavy‐and light‐chain bands in the nonreduced sodium dodecyl sulfate–polyacrylamide gel electrophoresis for recombinant chimeric IgA, dIgA and IgM suggests either an inability to assemble into heterodimers or that they are more sensitive to denaturation under these conditions. In all cases, other than IgG, which was purified as a single homogenous species, free kappa chain was present in the protein L–purified preparations (Supplementary figure 2e).

**Figure 1:**
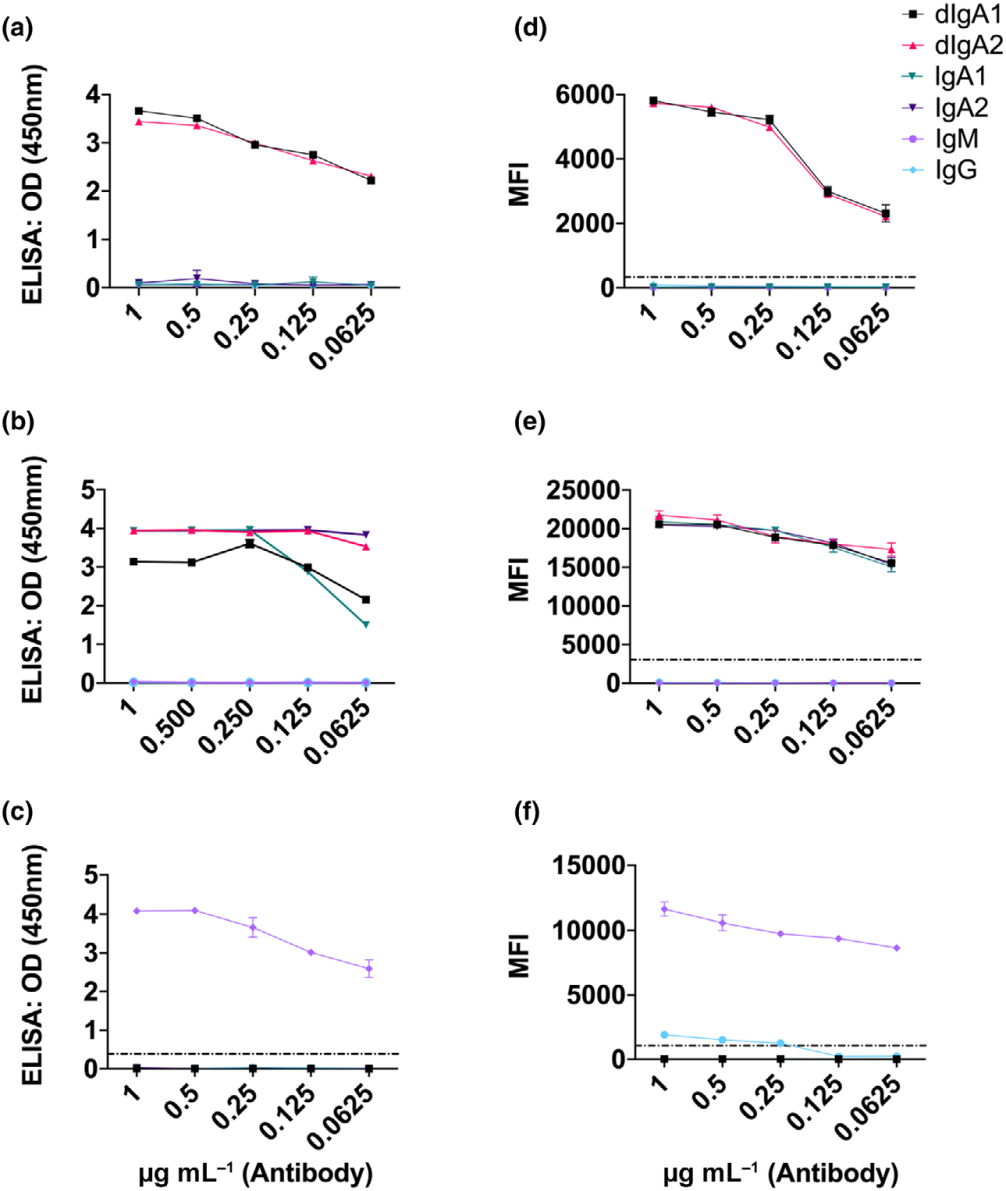
Detection of dimeric immunoglobulin A (dIgA), IgA and IgG antibodies using the ELISA and multiplex bead assay (MBA). Specificity of the ELISA to measure **(a)** dIgA, **(b)** IgA and **(c)** IgG to severe acute respiratory syndrome coronavirus 2 (SARS‐CoV‐2). Signals were measured at 450 nm. Data shown are two technical replicates for each isotype and assay. Specificity of the MBA to measure **(d)** dIgA, **(e)** IgA and **(f)** IgG to SARS‐CoV‐2. Signals were measured in mean fluorescence intensity units (MFI). Dotted lines indicate assay cut‐offs. Error bars represent standard deviations. OD, optical density.

Recombinant protein L–purified monoclonal antibodies were serially diluted and applied to each assay. Dose‐dependent responses to each monoclonal subclass were observed in the ELISA and MBA dIgA *via* interaction with CSC and detection with anti‐SC. No binding was observed for IgG, monomeric IgA or IgM monoclonals even at the highest concentration (1 μg mL^−1^; Figure 1a, d). The IgA MBA and ELISA showed similar sensitivity for both IgA and dIgA detection, and absence of nonspecific reactivity to IgG or IgM monoclonals (Figure 1b, e). Low‐level nonspecific reactivity to IgM monoclonals was observed at the highest concentration (1 μg mL^−1^) when measuring IgG responses in the MBA (Figure 1f). These results demonstrate the specificity of the ELISA and MBA for the isotype being tested. Interassay % coefficient of variation is a measure of the variance between runs of sample replicates on different plates, with scores less than 15 generally considered acceptable. In our hands we observed an interassay % coefficient of variation of 9.3 for the MBA and 8.1 for the ELISA. Expanding on this, we examined what effect the presence of other Ig subclasses would have on the sensitivity of dIgA measurements in both ELISA and MBA. In the presence and absence of convalescent plasma, a twofold serial dilution of CB6 dIgA was generated and mixed with each monoclonal Ig subclass (IgG, IgA and IgM) or a combination of all three Ig subclass at a fixed concentration (Supplementary figure 3a–d). No effect on the capacity of these assays to detect dIgA was observed.

To examine assay specificity, 200 pre–COVID‐19 plasma samples, collected prior to December 2019, were screened. Of the total 200 samples, two (ELISA) and three (MBA) samples were reactive to SARS‐CoV‐2 RBD and recorded a positive dIgA result, providing a specificity of 99.0% and 98.5%, respectively. Five (ELISA) and two (MBA) plasma samples recorded a positive result for IgA antibodies, providing a specificity of 97.5% and 99.0%, respectively. Three (ELISA) and four (MBA) plasma samples recorded a positive result for IgG antibodies providing specificity of 98.5% and 98%, respectively. These data indicate that both the ELISA and MBA provide high specificity for SARS‐CoV‐2–specific dIgA, IgA and IgG (Supplementary figure 4a–f and Table 1).

**Table 1:** Specificity of dIgA, IgA, IgG ELISA and MBA against SARS‐CoV‐2.

	Negative	False positive	Specificity	Total (*n*)
ELISA				
dIgA	198	2	99.0	200
IgA	195	5	97.5	200
IgG	197	3	98.5	200
MBA				
dIgA	197	3	98.5	200
IgA	198	2	99.0	200
IgG	193	4	98.0	200

Ig, immunoglobulin; MBA, multiplex bead assay; SARS‐CoV‐2, severe acute respiratory syndrome coronavirus 2.

ELISA and MBA performance was compared using a cross‐sectional community cohort consisting of 199 samples from patients infected with SARS‐CoV‐2. Samples were collected from patients who returned a PCR‐positive result and ranged from 0 to 442 days after confirmed PCR positivity. In all antibody measurements, the ELISA and MBA correlated significantly (*P*‐values for each correlation ≤ 0.001) with a strong positive linear relationship with *R* values of 0.81, 0.74 and 0.91 for dIgA, IgA and IgG, respectively (Supplementary figure 5a–c), suggesting that the MBA and ELISA are highly correlated for measuring dIgA, IgA and IgG antibodies to SARS‐CoV‐2.

### Temporal development of dIgA antibodies is short‐lived compared with IgA and IgG responses

To investigate when dIgA appears after exposure to SARS‐CoV‐2 and compare this with the development of IgA and IgG responses, we examined antibody levels over time in plasma samples from 125 patients. These were sourced from three cohorts before vaccination with PCR–confirmed SARS‐CoV‐2 infection (Supplementary table 1) where samples were collected (i) regularly from days 0 to 140 from individuals after diagnosis (Biomex cohort), (ii) from individuals presenting at a COVID‐19 testing clinic with PCR‐confirmed SARS‐CoV‐2 infection not requiring hospitalization (community cohort) and (iii) from patients admitted to the intensive care unit with severe COVID‐19 symptoms and were obtained between 2 and 440 days after PCR diagnosis (intensive sample cohort). All patients from the intensive cohort were hospitalized, with many requiring one or more of the following treatments: supplemental oxygen, extracorporeal membrane oxygenation or ventilation.

**Figure 2:**
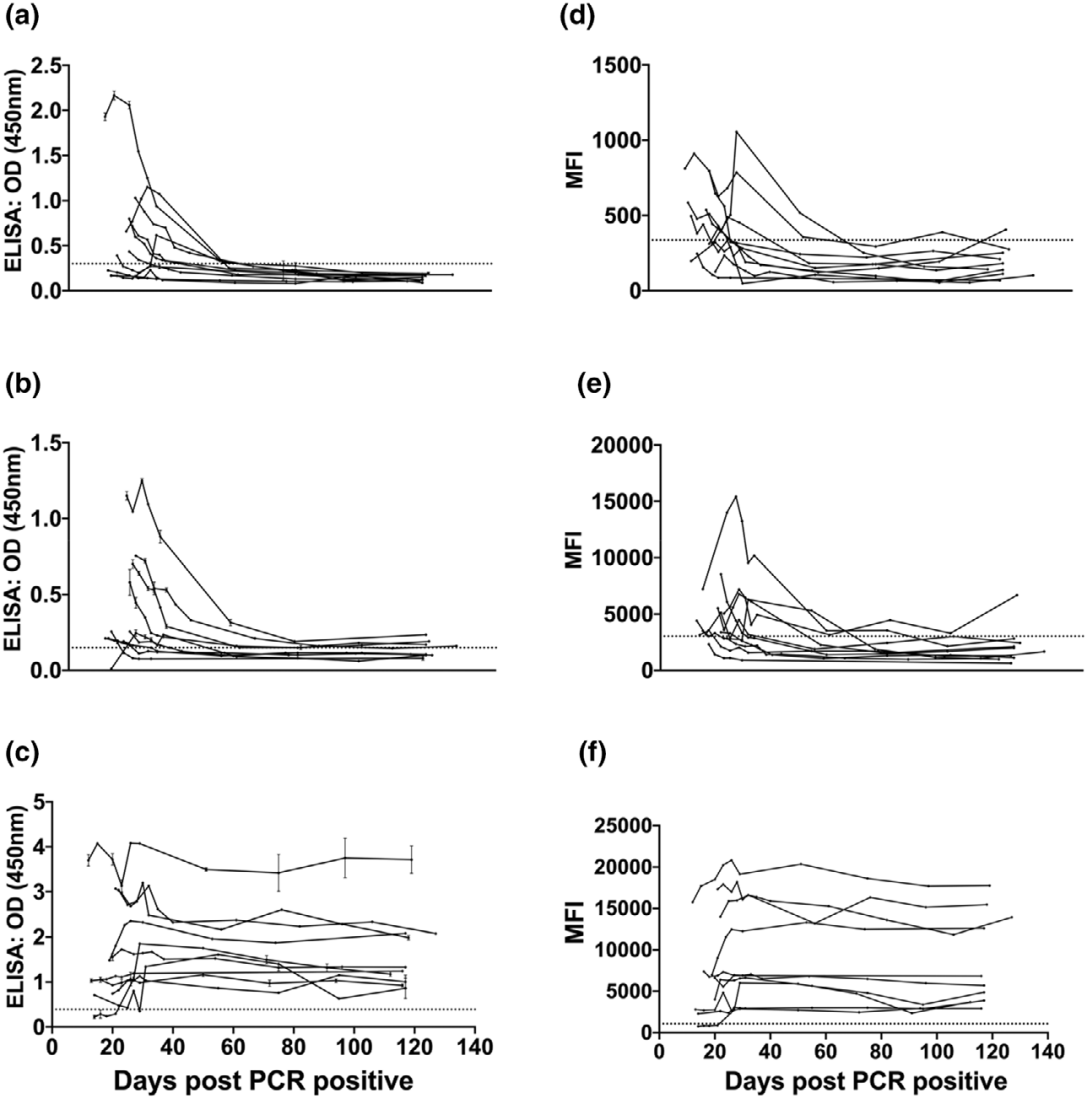
Distinct temporal dimeric immunoglobulin A (dIgA), IgA and IgG antibody profiles in individuals with mild severe acute respiratory syndrome coronavirus 2 (SARS‐CoV‐2) infection. Antibody profiles of dIgA, IgA and IgG responses 140 days after positive PCR result (*n* = 96 from 10 individuals). Data shown are one experimental replicate for each isotype and assay. The ELISA measuring **(a)** dIgA, **(b)** IgA and **(c)** IgG to SARS‐CoV‐2. Signals were measured at 450 nm. The multiplex bead assay (MBA) measuring **(d)** dIgA, **(e)** IgA and **(f)** IgG to SARS‐CoV‐2. Signals were measured in mean fluorescence intensity units (MFI). Dotted lines indicate assay cut‐offs. Error bars represent standard deviations. OD, optical density.

Temporal analysis of dIgA compared with IgG and IgA revealed that all patients sourced from the Biomex cohort had increasing amounts of dIgA within the first 3 weeks after PCR in both the dIgA‐MBA and the dIgA‐ELISA (Figure 2a, d). The levels of dIgA were higher than those of IgA (Figure 2b, e) but were short lived, with most patients having no dIgA detectable above assay background 45 days after positive PCR. By contrast, IgG levels were sustained and detected for at least 120 days after PCR diagnosis (Figure 2c, f).

### The temporal profile of the dIgA response is distinct from IgA and IgG in people with severe COVID‐19 infection

**Figure 3:**
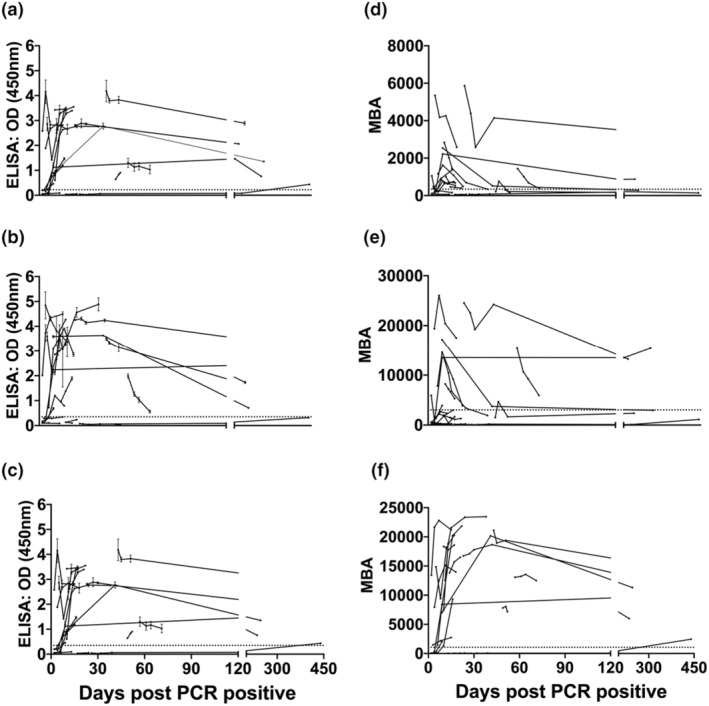
Analysis of dimeric immunoglobulin A (dIgA), IgA and IgG antibody responses to severe acute respiratory syndrome coronavirus 2 (SARS‐CoV‐2) in a longitudinal cohort of intensive care patients. Plasma samples (*n* = 74) from 20 individuals, obtained up to 450 days after PCR confirmation of SARS‐CoV‐2 infection. Data shown are one experimental replicate for each isotype and assay. The ELISA measuring **(a)** dIgA, **(b)** IgA and **(c)** IgG to SARS‐CoV‐2. Signals were measured at 450 nm. The multiplex bead assay (MBA) measuring **(d)** dIgA, **(e)** IgA and **(f)** IgG to SARS‐CoV‐2. Signals were measured in mean fluorescence intensity units (MFI). Dotted lines indicate assay cut‐offs. Error bars represent standard deviations. OD, optical density.

Samples in the intensive cohort presented a distinct and varied temporal profile compared with samples from patients with a mild SARS‐CoV‐2 infection. In the dIgA‐ELISA, 9/20 intensive patients showed increasing levels of dIgA antibodies throughout the sampling period, 6/20 had declining levels and 5/20 with low/absent antibody levels (Figure 3a–c).

Interestingly, the dIgA‐MBA showed that only 4/20 patients had low/absent dIgA antibody levels, 8/20 had declining levels and 8/20 showed increasing levels of dIgA antibodies throughout the sample period (Figure 3d–f).

### dIgA profile in the community cohort

**Figure 4:**
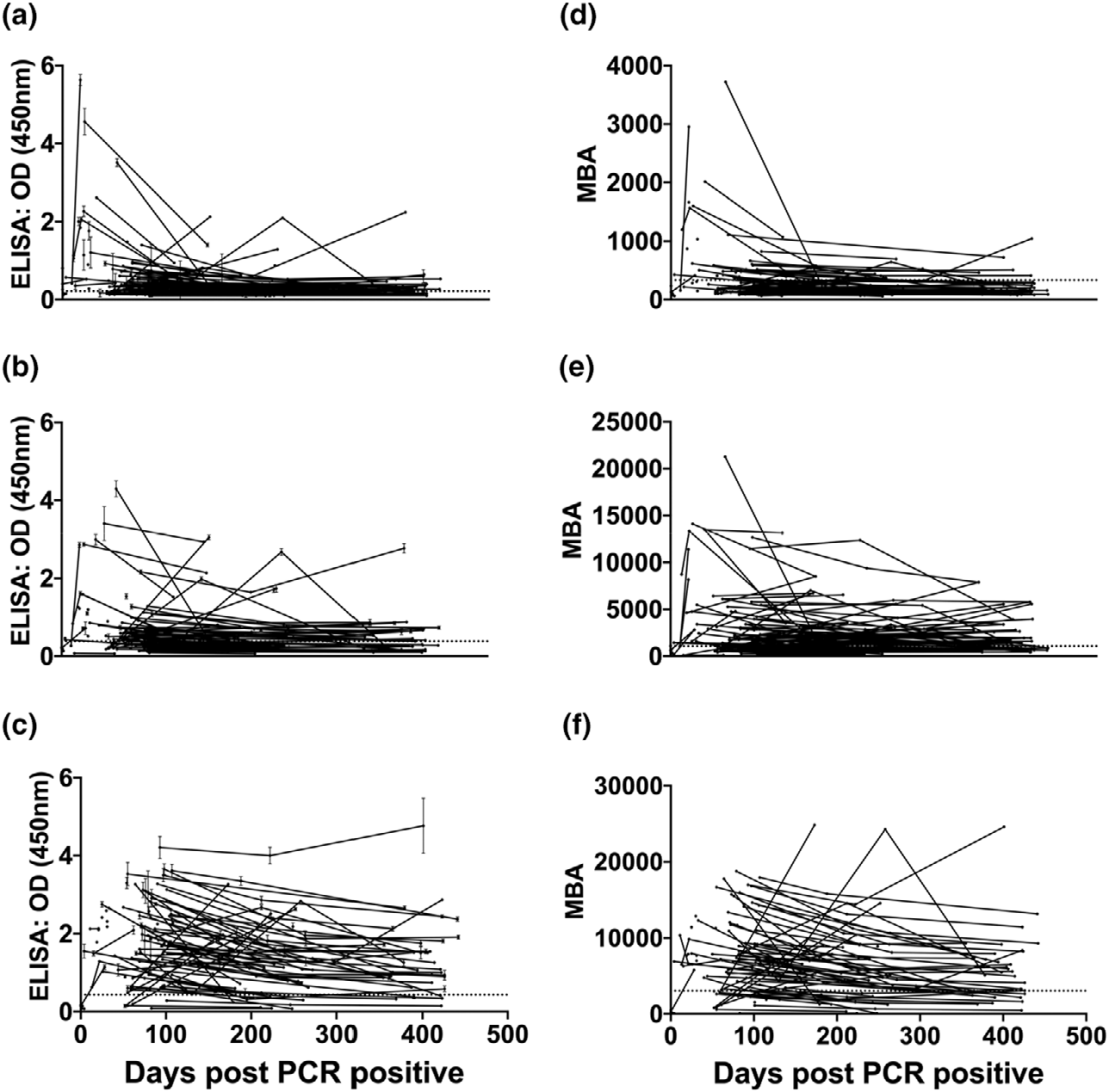
Analysis of dimeric immunoglobulin A (dIgA), IgA and IgG antibody responses to severe acute respiratory syndrome coronavirus 2 (SARS‐CoV‐2) in a cross‐sectional community cohort. Plasma samples (*n* = 199) obtained up to 500 days after PCR confirmation of SARS‐CoV‐2 infection. Data shown are one complete experimental replicate for each assay and isotype. The ELISA measuring **(a)** dIgA, **(b)** IgA and **(c)** IgG to SARS‐CoV‐2. Signals were measured at 450 nm. The multiplex bead assay (MBA) measuring **(d)** dIgA, **(e)** IgA and **(f)** IgG to SARS‐CoV‐2. Signals were measured in mean fluorescence intensity units (MFI). Dotted lines indicate assay cut‐offs. Error bars represent standard deviations. OD, optical density.

An analysis of dIgA, IgA and IgG responses to SARS‐CoV‐2 RBD within the community cohort was performed. The patients in this cohort had mild COVID‐19 symptoms and samples were collected between day 0 and up to 450 days after confirmed PCR. Our analysis revealed that dIgA responses are short lived compared with IgA and IgG responses, which persist for longer (Figure 4a–f). As anti‐human IgA secondary antibody reagents detect both IgA and dIgA, the early IgA levels detected could be dominated by dIgA, while later IgA detection may be dominated by monomeric IgA. The use of CSC to specifically detect dIgA allows the distinction of these two IgA responses and provides new insight into the development of mucosal immunity.

## DISCUSSION

SIgA protects mucosal surfaces by exploiting an active mechanism to transport polymeric immunoglobulins to effector sites within mucosal secretions. During an infection, plasma B cells in the submucosa secrete dIgA which is transcytosed across mucosal surfaces and released as SIgA. Previously, it has not been possible to measure dIgA in plasma because of the high degree of cross reactivity of secondary antibodies with both dIgA and monomeric IgA. Here, we describe two assays that allow the specific measurement of dIgA amenable to large serological studies and allow for the first time an examination of the temporal production of dIgA. The dIgA‐ELISA and dIgA‐MBA correlate significantly in measuring dIgA, IgA and IgG antibodies, with *R* values of 0.81, 0.74 and 0.91, respectively (*P*‐values < 0.001 in all cases).

In both assays, dIgA specific for SARS‐CoV‐2 RBD in patients with uncomplicated COVID‐19 with mild symptoms is short lived, first detected at 12 days after PCR diagnosis but undetectable in 80% of cohort patients by day 25. This is most likely a result of homing of B cells that produce dIgA through the bloodstream to mucosal surfaces and the transport of dIgA across mucosal surfaces where it is converted to SIgA. However, it could also be a result of a transitory burst of IgA and plasma blasts or variable inhibition by increasing levels of RBD‐IgG. Regardless, this is the first study to specifically describe assays that allow the monitoring of the dIgA response in an infection and provides new insights into the role of dIgA in immunity to respiratory and other mucosal infections. An assay to specifically detect dIgA in the periphery will be valuable in studies of vaccine responses where mucosal immunity is desired and will complement measurement of SIgA in mucosal secretions.

In contrast to patients presenting with mild COVID‐19 where dIgA was detected at lower levels and for shorter periods (0–25 days), patients who had severe COVID‐19 had detectable dIgA up to 250 days after PCR diagnosis. It is unknown why dIgA would be detectable for such an extended period in severe COVID‐19. One mechanism could be related to prolonged virus infection and antigen expression causing continued B‐cell stimulation. Alternatively, severe COVID‐19 infection may impair transcytosis of dIgA across mucosal surfaces resulting in an accumulation of dIgA in blood, and lack of SIgA at the mucosa, which could in turn impair viral clearance, contributing to severe COVID‐19. Other mechanisms that could be contributing to dIgA levels for such an extended period include liver disease, microbial dysbiosis and intestinal disease.^10^, ^11^, ^12^, ^13^, ^14^

Within the Biomex cohort, the ELISA levels of dIgA and IgA are similar across four individuals, whereas the MBA shows a clear differentiation in the levels of dIgA and IgA. Overall, the MBA had slightly better resolution between dIgA and IgA responses and allowed simultaneous detection of IgG, IgA and dIgA within the same reaction, making it amenable to high‐throughput screening on samples where only small volumes are available.

Studies have suggested that individuals who have had mild or asymptomatic disease may produce lower levels of antibody, which dissipate faster over time than that in those with moderate to severe COVID‐19.^15^ To investigate this, we analyzed dIgA, IgA and IgG across two cohorts with longitudinal sampling and compared the difference in antibody response in patients over time. Notably, in individuals hospitalized with severe COVID‐19, we observe higher dIgA levels, which appear to be present for longer periods, and contrasts with the short‐lived responses seen in individuals with mild and self‐limiting infections. The Biomex cohort (Figure 2) consisted of patients that are considered to have had mild symptoms and all patients had dIgA antibody levels that are significantly lower than IgG. We observe high dIgA signals in patients from the intensive unit cohort in both the ELISA and MBA which appear to be short lived (Figure 3). Contrastingly, IgG antibodies appear to last longer and fluctuate less than dIgA levels in patients with either mild or severe COVID‐19 symptoms. It is important to note that the time scale of the intensive unit cohort is up to 440 days, which is significantly longer than the time scale of the Biomex cohort of 107 days. While dIgA showed a distinct longitudinal profile compared with IgG, it appears to follow a similar longitudinal profile as IgA (Figure 3). These results demonstrate that a component of the IgA response being measured using conventional serology could be dIgA.

Two patients within the intensive cohort had no detectable dIgA, IgA or IgG antibodies in both the ELISA and MBA. A further two patients had no IgA or dIgA antibodies by the ELISA but were positive for these isotypes by the MBA. This suggests that the MBA method is more sensitive than the ELISA method. The prevalence of IgA deficiency varies depending on ethnic background, with frequency estimates in the United States ranging from 1 in 200 to 1 in 1000. IgA deficiency is more commonly found in Whites and affects the production of both monomeric IgA and dIgA responses.^16^ It is possible these patients suffered from a primary or secondary immunodeficiency, preventing them from generating a strong antibody response. Unfortunately, any clinical information on comorbidities of the patients in the intensive cohort is not available. It is possible that patients with no detectable level of dIgA antibodies were IgA deficient as no detectable level of IgA was detected either. All current serological tests measure total IgA. Despite dIgA being a component of total IgA, there are differences in the temporal profile during infection. The results presented here demonstrate that further investigation is needed into the roles of plasma dIgA as a biomarker of early (acute) infections, and as a measure of mucosal immune responses.

### Strengths/limitations/specifications

Prospective longitudinal study samples would be beneficial to further assess the sensitivity of dIgA responses in both the MBA and ELISA. The MBA can be performed at medium‐ to high‐throughput and incorporated into existing serological disease screening panels. However, the MBA requires specialized equipment that is expensive and restricted to laboratory settings. By contrast, the ELISA does not necessarily require instrumentation and is more amenable to use in resource‐constrained settings. The major limitation to both tests used here, which are based on the detection of SARS‐CoV‐2 RBD‐specific responses, is that they cannot discriminate between infection and vaccination. However, the assay could be easily converted to detect nucleoprotein‐specific responses indicative of infection. Serosurveys conducted in the postvaccine era are likely to estimate the combined level of exposure to SARS‐CoV‐2 and vaccination, but do not directly indicate the level of immunity to SARS‐CoV‐2 infection. The dIgA assay using CSC reagents can be adapted to detect the production of dIgA to any pathogen and has previously been used in hepatitis C, hepatitis E and hepatitis A viruses.^8^ The quantitative assays described here allow, for the first time, a method to measure dIgA and further understand its role in the immune system.

## METHODS

### CB6 monoclonal antibodies

Antibodies and proteins. Recombinant synthetic chimeric IgA1 and IgA2 heavy‐chain sequences were constructed by joining the CB6 variable domain (amino acids 1–137) to the CH1 domain (amino acids 161–516) of IgA1 (J00220) or to the CH1 domain (amino acids 161–501) of IgA2 (J00221) or to the CH1 domain (amino acids 139–590) of IgM (AAS01769) *via* a *Bsp*E1 restriction site and cloning into a pcDNA3 vector with an N‐terminal TPA leader sequence (Thermo Fisher, Waltham, MA, USA). Recombinant synthetic J chain DQ884395 (amino acids 22–175) was expressed in frame with the tissue plasminogen activator leader sequence *via* a pcDNA3 expression vector (Thermo Fisher). Recombinant chimeric IgA1 and IgA2 were expressed in Expi 293 cells (Thermo Fisher Scientific, Waltham, Massachusetts, USA) by co‐transfection of the CB6 kappa chain and CB6‐IgA1 or CB6‐IgA2 heavy‐chain sequence. dIgA1 and dIgA2 were expressed by co‐transfecting CB6 kappa light chain and CB6‐IgA1 or CB6‐IgA2 heavy‐chain sequence and pcDNA3‐J (Thermo Fisher Scientific) using equivalent amounts of DNA. After 4 days, supernatant fluid was collected, clarified and either used directly or purified using 45% ammonium sulfate (Thermo Fisher Scientific)  and size exclusion chromatography on a Superose 6 Increase 10/60 GL column (ÄKTA pure). Proteins were analyzed on sodium dodecyl sulfate–polyacrylamide gel electrophoresis and visualized with Coomassie blue (Thermo Fisher Scientific). The construction and expression of recombinant synthetic CSC protein have been previously.^8^, ^9^ The following reagents were sourced from commercial suppliers: Protein L‐agarose (Thermo Fisher Scientific); SARS‐CoV‐2 RBD (GenScript catalog number Z03483‐1, Piscataway, New Jersey, USA); BetaCov/Australia/Vic/01/2020 (NISBC) and 5D protein stabilizer (Abacus dx catalog number 5D82411B, Queensland, Australia).

### Sample populations

All human specimen materials were considered infectious and hazardous and handled using standard biosafety procedures. Specimens were frozen at −80°C for longer‐term storage and tested as soon as possible after thawing. Prior to testing, frozen samples were brought to room temperature slowly and gently mixed. Samples containing visible particulate matter were clarified by centrifugation before testing. Plasma samples, including COVID‐19 acute/convalescent samples, were obtained from the Alfred Hospital, Biobank, Australian Red Cross Lifeblood and Biomex (Supplementary table 1). All plasma used in this study were collected before vaccination for SARS‐CoV‐2.

The Lifeblood negative cohort consisted of 200 samples of naïve plasma collected from individuals before SARS‐CoV‐2 infection. The Biomex cohort consisted of plasma samples obtained from Biomex, Germany, from 10 individuals confirmed of having a SARS‐CoV‐2 infection. Individuals were sampled longitudinally. The intensive cohort consisted of plasma samples from individuals admitted to an intensive care unit and confirmed to have SARS‐CoV2 by PCR. Individuals were sampled longitudinally. Finally, the community cross‐sectional samples were sourced from COVID testing clinic patients. These intensive and community cohorts from individuals with a PCR‐confirmed diagnosis of COVID‐19 were enrolled to the Alfred Health COVID‐19 Biobank (Alfred Health Human Research and Ethics Committee Number 182/20).

### MBA

The MBA described in Mazhari *et al*.^17^ was used with the following amendments. Prior to the addition of antigen‐coupled beads, the plasma was incubated with CSC reagent at a concentration of 4 μg mL^−1^ in PBT (1% bovine serum albumin 1× phosphate‐buffered saline + 0.05% Tween 20 (all items from Sigma, St Louis, Missouri, USA) for 2 h at room temperature. Prior to the addition of F(ab′) anti‐mouse IgG (H + L) phycoerythrin‐conjugated secondary antibody (Thermo Fisher Scientific), an anti‐CSC antibody at a concentration of 0.5 μg mL^−1^ was added to the mix and incubated for 1 h at room temperature (Supplementary figure 1c). CB6 monoclonal experiments were performed as above, but with the plasma dilution step replaced with the dilution of CB6 monoclonal antibody (made in house). The construction and expression of recombinant synthetic CSC protein has been previously described in Mohd Hanafiah *et al*.,^8^ Drummer *et al*.,^9^ Roe *et al*.,^18^ Braathen *et al*.^19^ A pool of SARS‐CoV‐2–positive patients and a negative control are included on each plate. All other steps are as described.^17^ Cut‐off values were calculated using the mean + 3 standard deviations of the negative cohort. For IgA, IgG and IgM MBA plasma samples were added to antigen‐coupled beads and RBD‐specific IgA, IgG and IgM were captured by a phycoerythrin‐conjugated anti‐human IgG Fc secondary antibody (Abacus dx catalog number JIR709116098) (Supplementary figure 1d).

### ELISA

ELISA plates (Nunc MaxiSorp 96‐well flat‐bottom; Thermo Fisher Scientific, Waltham, Massachusetts, USA) were coated with recombinant S‐RBD at a concentration of 1 μg mL^−1^ and blocked with 1.5% bovine serum albumin in phosphate‐buffered saline. For the dIgA‐ELISA, plasma samples were diluted (1:100) in ELISA diluent buffer (1% bovine serum albumin 1× phosphate‐buffered saline + 0.05% Tween 20) in the presence of CSC reagent (4 μg mL^−1^), and then added to an S‐RBD precoated plate for 2 h at 37°C. Following six washes with ELISA wash buffer (1× phosphate‐buffered saline + 0.05% Tween 20), a mouse anti‐CSC (6D5) antibody (at a concentration of 0.5 μg mL^−1^) was added and incubated for 1 h at 37°C and followed by an anti‐mouse IgG‐horseradish peroxidase (HRP) antibody (Supplementary figure 1a). For IgA, IgG and IgM ELISA, plasma samples were added and incubated in the well precoated with recombinant S‐RBD. SARS‐CoV‐2 RBD‐specific IgA, IgG and IgM were captured and then detected by anti‐human IgA‐HRP, anti‐human IgG‐HRP or anti‐human IgM‐HRP antibody (Supplementary figure 1b). CB6 monoclonal experiments were performed as above, but with the plasma dilution step replaced with the dilution of CB6 monoclonal antibody. The following reagents were sourced from commercial suppliers: SARS‐CoV‐2 RBD (GenScript catalog number Z03483‐1); CSC (GenScript customized); Mouse anti‐SC 6D5 (GenScript customized); anti‐mouse IgG HRP: goat anti‐Mouse IgG (H + L) highly cross‐adsorbed secondary antibody, HRP (Thermo Fisher Scientific catalog number A16078); anti‐human IgA HRP: goat anti‐human IgA HRP (Southern Biotech catalog number 2050–05; Birmingham, AL, USA); anti‐human IgM: goat pAb to huIgM HRP (Abcam catalog number ab97205; Cambridge, UK). An ELISA calibrator was used on each plate. Cut‐off values were calculated using the mean + 3 standard deviations of the negative controls.

### Statistical methods

Quality control of raw data from the MBA was performed using the QC functionality of the covid ClassifyR R Shiny web application (https://shaziaruybal.shinyapps.io/covidClassifyR). Data were analyzed using Prism version 9.02 using the two‐tailed nonparametric Spearman correlation test with 95% confidence intervals.

## AUTHOR CONTRIBUTIONS

**Zihui Wei:** Conceptualization; data curation; formal analysis; investigation; writing – original draft; writing – review and editing. **Fiona Angrisano:** Conceptualization; data curation; formal analysis; funding acquisition; investigation; writing – original draft; writing – review and editing. **Emily M Eriksson:** Data curation; methodology; writing – review and editing. **Ramin Mazhari:** Data curation; methodology; writing – review and editing. **Huy Van:** Methodology; resources. **Shuning Zheng:** Methodology; resources. **Rob J Center:** Resources; writing – review and editing. **Irene Boo:** Resources; data curation; investigation; writing – review and editing. **James McMahon:** Resources. **Jillian Lau:** Resources. **Nicholas Kiernan‐Walker:** Investigation; writing – review and editing. **Shazia Ruybal‐Pesántez:** Software; writing – review and editing. **Ivo Mueller:** Supervision. **Leanne J Robinson:** Supervision. **David A Anderson:** Methodology; writing – review and editing. **Heidi E Drummer:** Conceptualization; funding acquisition; supervision; writing – original draft; writing – review and editing.

## CONFLICT OF INTEREST

The authors declare there are no conflicts of interest.

## ETHICS APPROVAL

This study was conducted in concordance with the principles of the Declaration of Helsinki of the World Medical Association and approved by local human research ethics committees. Ethics approval was granted for this work by Alfred HREC (296/20: Development of serological tests for COVID‐19). Pre‐pandemic negative control sera were provided by Australian Red Cross Lifeblood (Material supply agreement 20‐08 Vic‐05). All samples were de‐identified prior to testing. This report includes an analysis of stored samples and data from those studies. No additional samples were collected for the current study.

## Supporting information


Supplementary figure 1
Supplementary figure 2
Supplementary figure 3
Supplementary figure 4
Supplementary figure 5
Supplementary table 1

## Data Availability

All data in this manuscript will be provided upon email request to the corresponding author.
