# Supplementary material for: Serological assays to measure dimeric IgA antibodies in SARS‐CoV‐2 infections
Source: Immunol Cell Biol. 2023 Aug 18;101(9):857–66. doi: 10.1111/imcb.12682 (PMC10952984; doi:10.1111/imcb.12682)
Supplement: Supplementary file 1 — Supplementary figure 1 Supplementary figure 2 Supplementary figure 3 Supplementary figure 4 Supplementary figure 5 Supplementary table 1 [file IMCB-101-857-s001.docx]

**
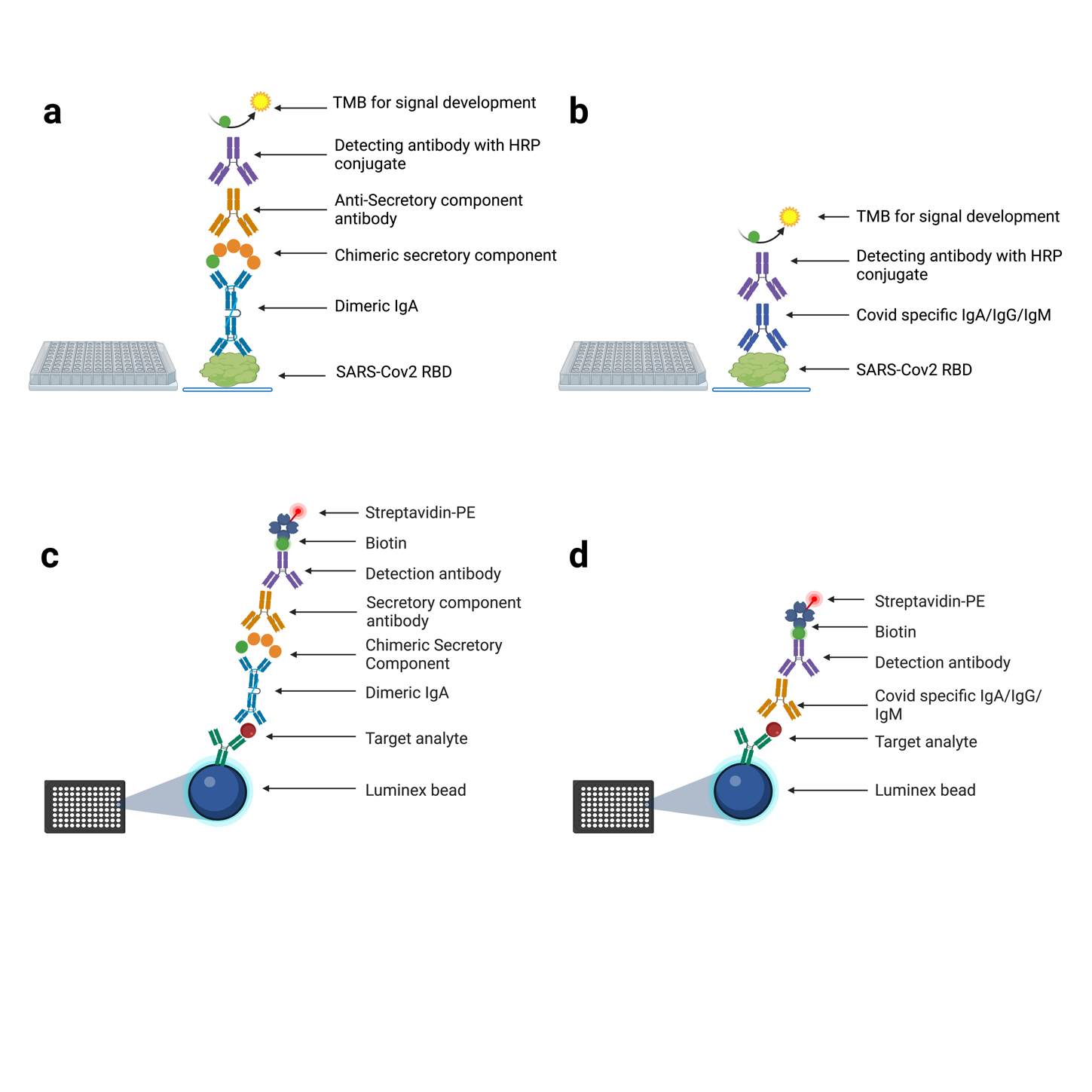
­­**

**Supplementary figure 1.** Schematic of assays. Enzyme linked immunosorbent assay for detection of dIgA **(a)** and IgG, IgA or IgM **(b)**. Multiplex bead array assay for detection of dIgA **(c)** and IgG, IgA or IgM **(d)**. ­­

**
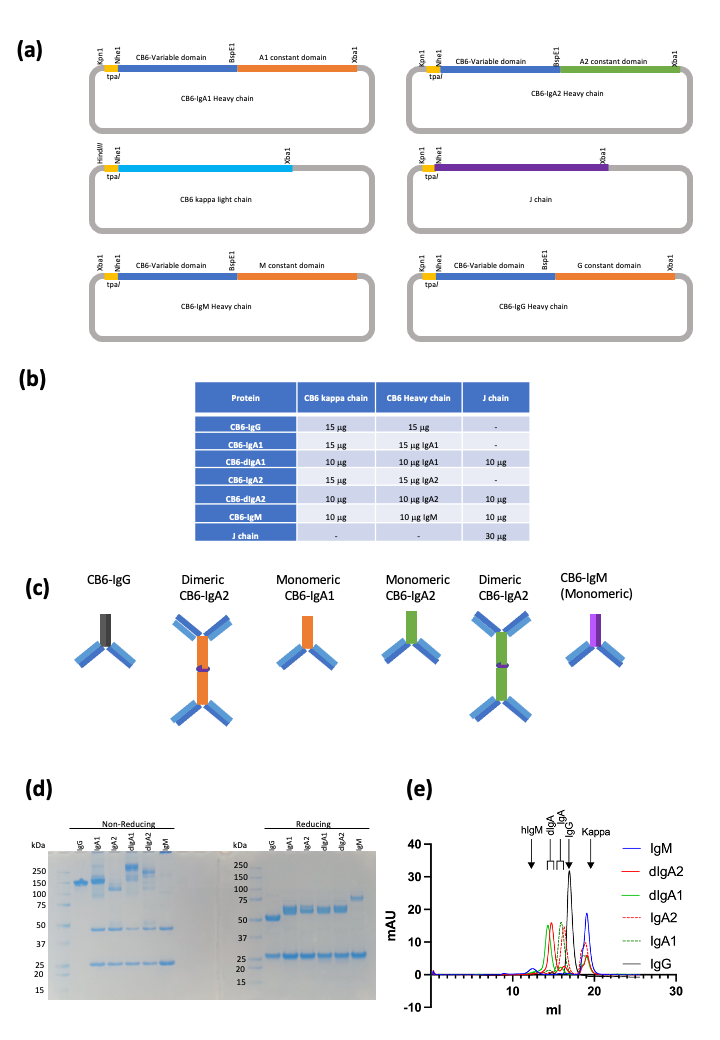
**

**Supplementary figure 2. dIgA production of CB6 monoclonals**. Production of control CB6 antibodies. **(a)** Schematic drawing of heavy and light chain clones used to generate recombinant monoclonal CB6 antibody of different isotypes. **(b)** Amount of plasmid DNA used per transfection to produce recombinant monoclonal CB6 antibodies in 293 F cells. **(c)** Schematic of different CB6 isotypes. **(d)** Coomassie stained SDS-PAGE of Protein L purified monoclonal antibodies under non-reducing (left panel) and reducing (right panel) conditions. **(e)** Size exclusion chromatopgraphy of protein L purified proteins.


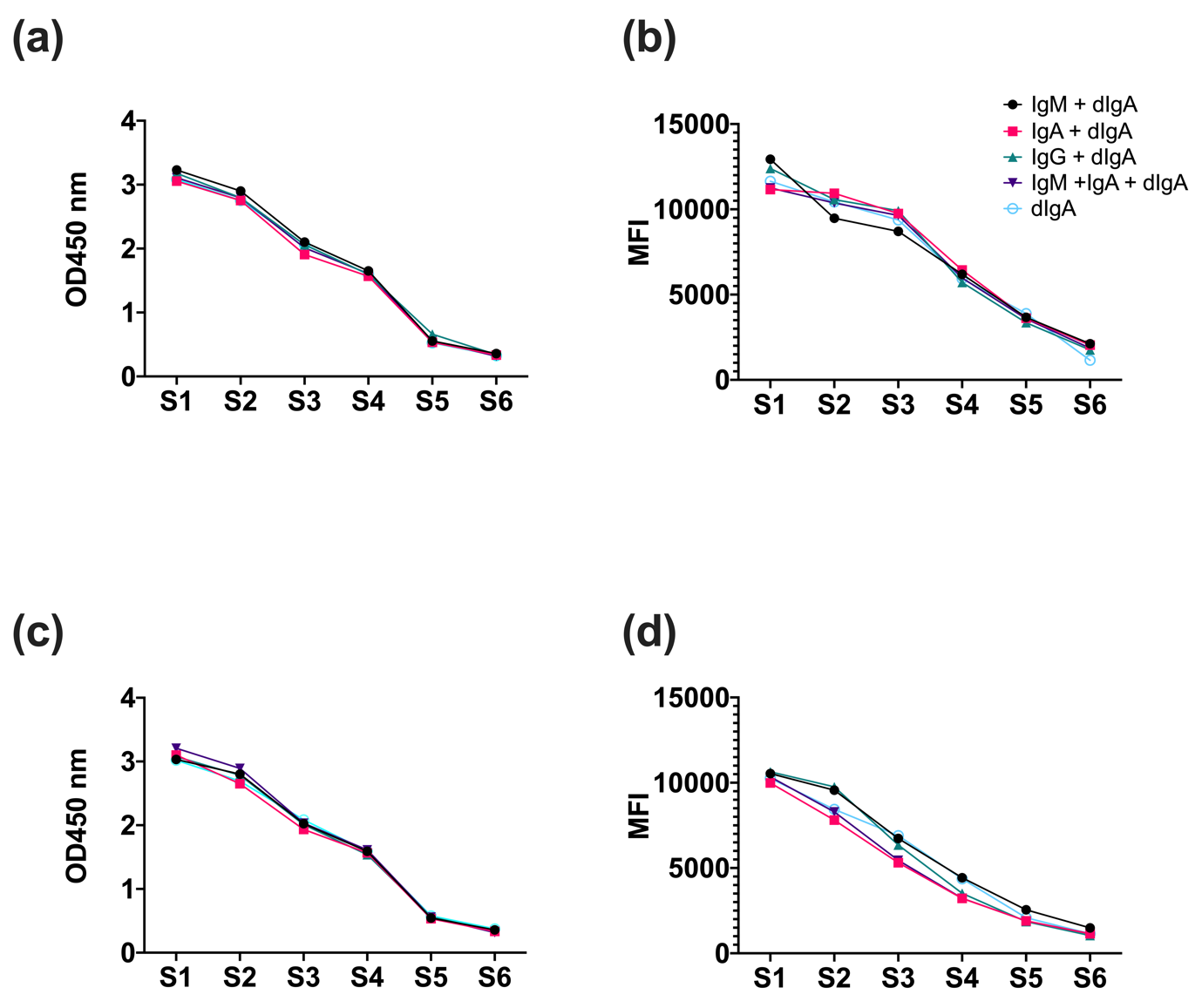


**Supplementary figure 3. Specificity of ELISA and MBA to measure dIgA.** Two-fold serial dilution of dIgA monoclonal to a fixed amount of each monoclonal Ig subclass (IgG, IgA, IgM) and combination of all 3 Ig subclasses. Serial dilutions performed in the absence **(a)**, **(b)** and presence **(c)**, **(d)** of pre-covid plasma. Data shown are one experimental replicate. ELISA signal measured at 450 nm. MBA signal measured in Mean fluorescence Intensity Units (MFI).

***
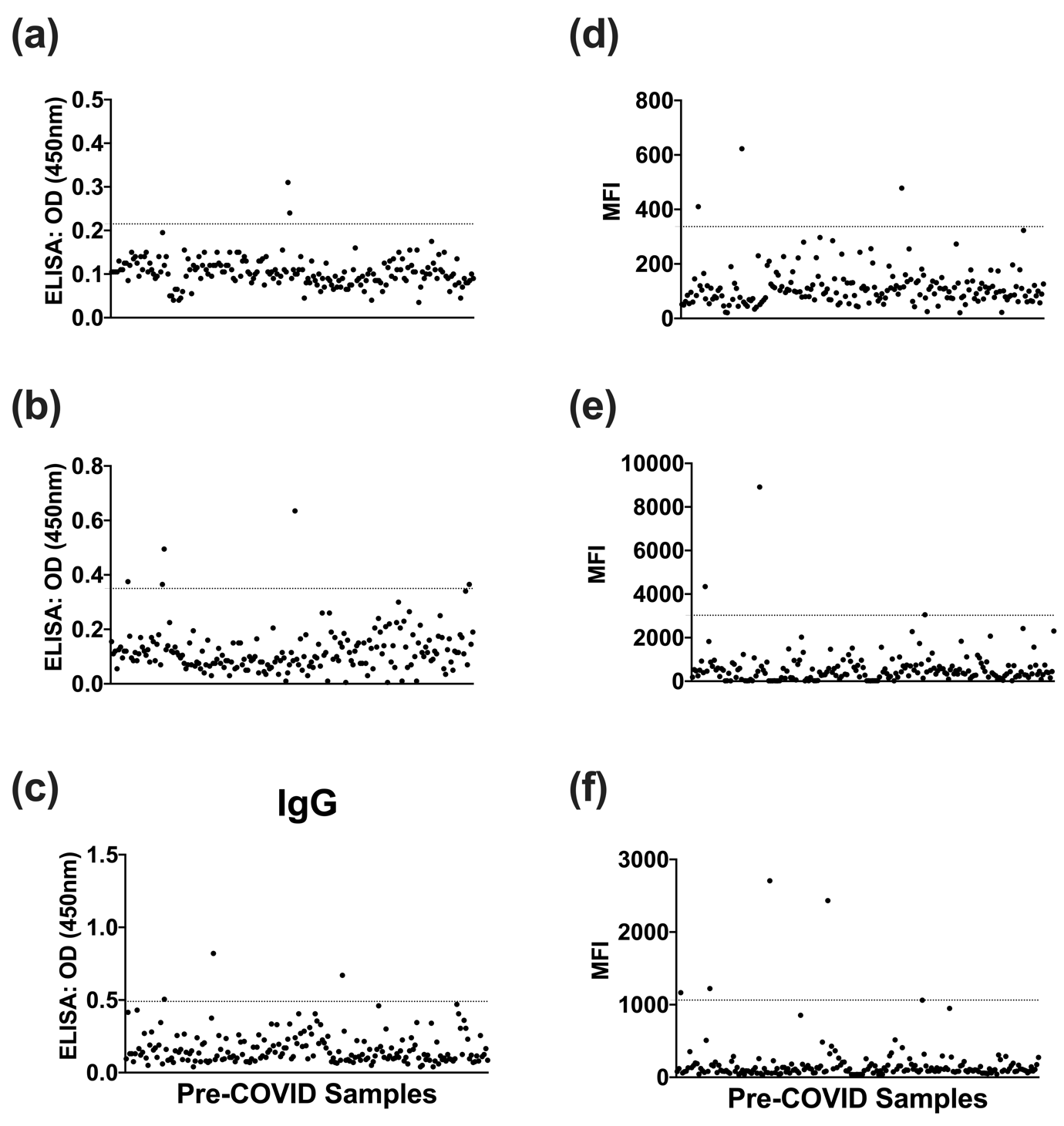
***

**Supplementary figure 4. Sensitivity of ELISA and MBA to measure dIgA, IgA and IgG to SARS-CoV-2.** Scatterplot of pre-covid samples n = 200. Specificity of ELISA to measure dIgA **(a)**, IgA **(b)** and IgG **(c)** to SARS-CoV-2. Signals were measured at 450 nm. Specificity of MBA to measure dIgA **(d)**, IgA **(e)** and IgG **(f)** to SARS-CoV-2. Data shown are one experimental replicate for each assay and isotype. Signals were measured in Mean Fluorescence Intensity units (MFI). Dotted lines indicate assay cut offs.


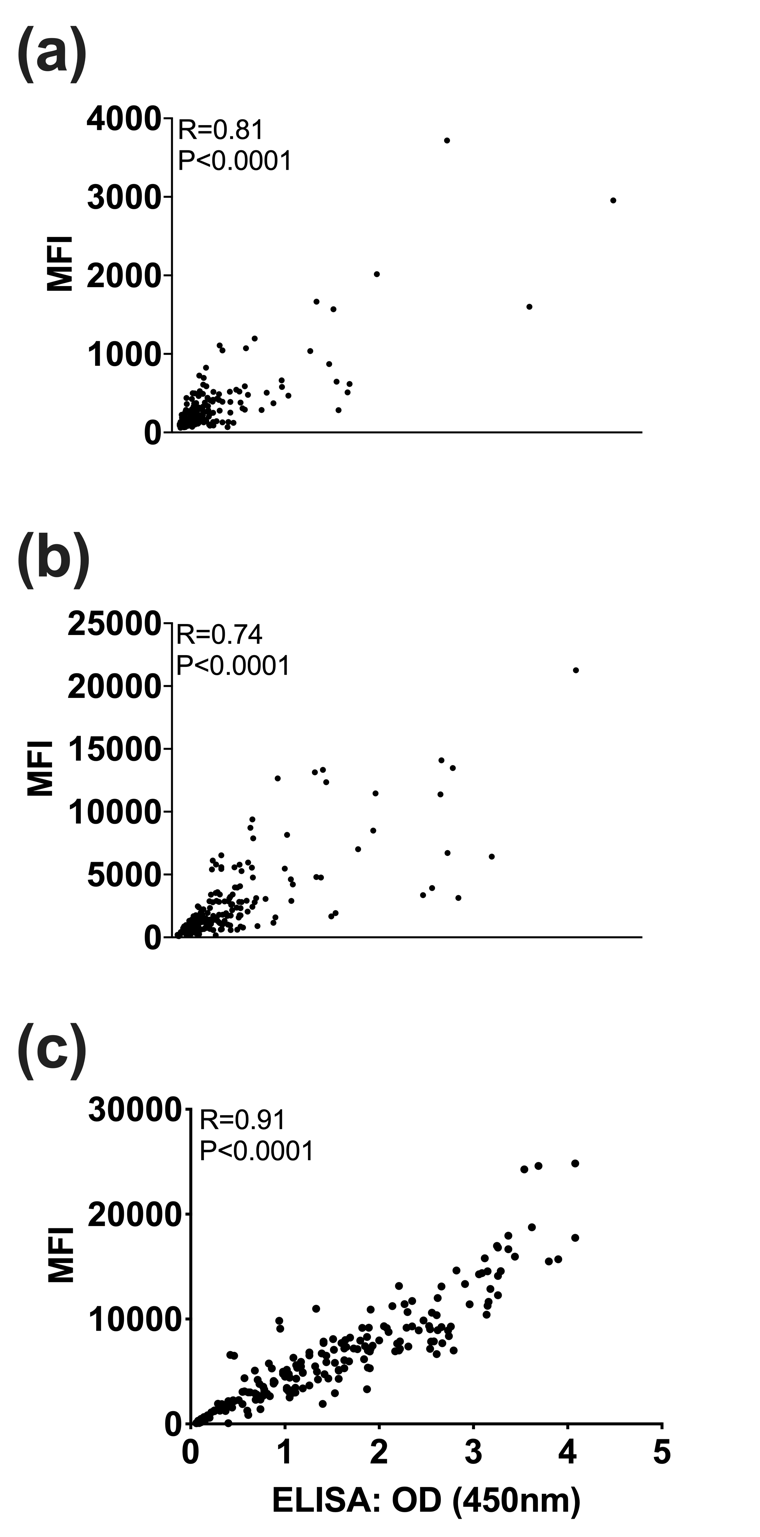


**Supplementary figure 5.** Correlation between ELISA and MBA when measuring **(a)** dIgA, **(b)** IgA and **(c)** IgG antibodies, sample n = 200. Data shown are one experimental replicate for each assay and isotype. Pearson’s R values 0.81, 0.74 and 0.91 observed respectively, *P*-values < 0.001.

**Supplementary table 1.** Study background and participants

| Cohort Name | Plasma Sample # | Description | Type of Study |
| --- | --- | --- | --- |
| Biomex | 96 (n = 10) | Subjects with mild COVID-19 symptoms | Longitudinal |
| Intensive | 74 (n = 20) | Subjects with severe COVID-19 symptoms and in ICU | Longitudinal |
| Community | 199 | Subject with mild symptoms from the community | Cross-sectional |
| Life Blood | 200 | COVID-19 negative samples | Pre-pandemic |
